# Comparison between Clinically Used Irregular Fields Shielded by Cerrobend and Standard Lead Blocks

**Published:** 2015-06-01

**Authors:** A. R. Farajollahi, F. Bouzarjomehri, M. Kiani

**Affiliations:** 1 Professor of Medical Physics, Tabriz University of Medical Sciences, Tabriz, Iran; 2 Professor of Medical Physics, Shahid Sadoughi University of Medical Sciences, Yazd, Iran; 3 MSc of Medical Physics, Tabriz University of Medical Sciences, Tabriz, Iran

**Keywords:** Shield, Cerrobend, Lead block, Dosimetry, Radiation therapy, TLD dosimetry

## Abstract

**Introduction:**

In radiation therapy centers across Iran, protection of normal tissues is usually accomplished by either Cerrobend or lead block shielding. In this study, the influence of these two shielding methods on central axis dose distribution of photon beam a Cobalt unit was investigated in clinical conditions.

**Materials and Methods:**

All measurements were performed for 60Co γ-ray beams and the Cerrobend blocks were fabricated by commercial Cerrobend materials. Standard lead block shields belonged to Cobalt unit. Data was collected through a calibrated ionization chamber, relative dosimetry systems and a TLD dosimetery.

**Results:**

Results of the percent depth dose (PDD) measurements at depths of 0.5, 1, 5, 10, 15 and 20 cm for 23 different field sizes of patients with head and neck cancer showed no significant differences between lead and Cerrobend shielding methods. Measurement results of absolute dosimetry in depths of 1.5, 3, 5, 7, 10 and 12 cm also showed no significant differences between these two shielding methods. The same results were obtained by TLD dosimetry on patient skin.

**Conclusion:**

Use of melt shielding methods is a very easy and fast shield-making technique with no differences in PDD, absolute and skin dose between lead and Cerrobend block shielding methods.

## Introduction

The goal of radiotherapy is to deliver an accurate dose to cancerous tissues and simultaneously avoid unnecessary dose to normal tissues. Therefore, it is essential that the field shaping be as perfectly individualized for patients as possible. Standard blocks cannot be used for all patients. Nowadays, the most common system for customized beam shaping uses a low melting point alloy, called Cerrobend[1-4] (Cerron Metal Products Company, Bellefonte, PA), also known as Lipowitz’s metal[5]. The standard lead blocks are made in cubic, pyramidal and cylindrical shapes with thicknesses that are proportional to the photon energy[6, 7]. In modern radiotherapy, radiation fields are created by Multileaf Collimator (MLC) with desired shape and are suitable for IMRT (intensity modulated radiotherapy) treatment planning[8]. In our country, use of an accelerator with an MLC collimator is limited; however, Cerrobend shielding method with an automatic cutter system is common. In casting method, the use of Cerrobend alloy is common due to its low melting point, high attenuation coefficient of photon beam, non-toxicity and reduction of Bremsstrahlung ray by electron beam[9]. Thickness of shielding block is determined by treatment beam energy. Cerrobend blocks are normally fabricated in molding room using a Styrofoam cutting system, while considering beam energy[5].

The purpose of the present study was to investigate dose distribution after using these two methods of shielding in clinical conditions. In addition to the advantage of easy and fast fabrication, individual patient shield reduces operational errors. In this study, Cerrobend and lead blocks shielding methods were compared in relation to dose distribution of photon beams of ^60^Co.

## Material And Methods

This study was performed using a ^60^Co unit (Phoenix, Nordiac, Canada) in Shahid Ramazanzade Radiotherapy Center, Yazd, Iran. Block shields were produced through two methods of Lead and Cerrobend melting. Fixed lead blocks were in the form of cubes, cylinders and pyramids with a thickness of 5 cm. Cadmium-free Cerrobend alloy blocks contained 50% bismuth, 13% tin and 32% lead. For block-shaped fields, the outlines of clinical irregular fields were transferred to a computer and in a process, Styrofoam molds were cut automatically for Cerrobend blocks (PAR Scientific Model ACD-4MK4, Denmark). Shield was positioned just outside the field at its inferior border. It was designed with steep edges in order to accommodate beam divergence. The field size data of 23 patients with head and neck cancers, who were treated by a Cobalt unit in 2012, were selected randomly. Scanditronicx-Wellhofer Farmer type ionization chamber (FC65-G) with an inner diameter of 6.2 mm and an active volume of 0.65 cm^3^ and Dose-1 Electrometer were used. All measurements were achieved by the detector with its central axis set perpendicular to the beam axis. The detector was also positioned at isocentric beam at 1.5, 3, 5, 7, 10 and 12 cm of water depth (SSD= 80cm) in a mini phantom with dimensions of 30× 30 × 30 cm. All measurements were achieved for 23 irregular fields which were shielded by Cerrobend and lead blocks.

Percent depth dose (PDD) data were collected by RFA-300 Plus water phantom, Scanditronicx-Wellhofer Omni Pro software and a diode detector at a range of depths from surface-to-depth of 30 cm for 23 irregular fields of patients who were irradiated by a Cobalt unit. Fields were limited using both Cerrobend and lead blocks. 

Skin dose and maximum depth dose were measured by LiF: Mg, Ti (TLD-100) thermoluminescent dosimeter (3×3×0.9 mm), using a TLD Rexon reader (made in America). 

## Results

**Figure 1:**
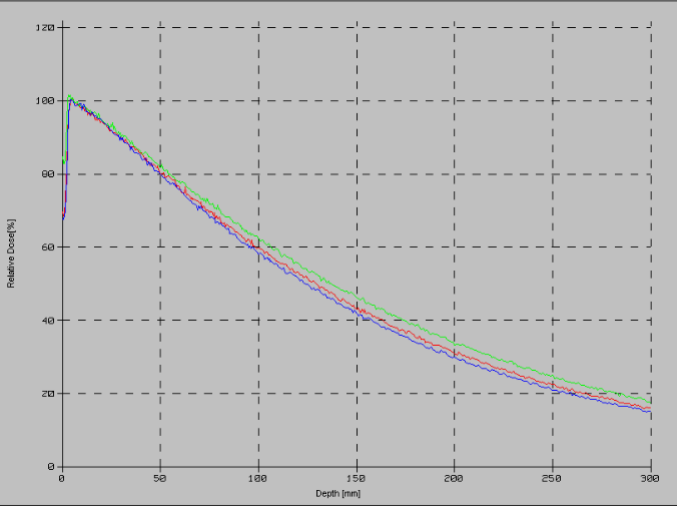
PDD Curves of the Fields Shielded by Lead Block (red), Cerrobend Block (blue) and without Shield (green). These curves were plotted by Omni Pro software of the water phantom.

Table 1 shows mean values and standard deviations of percent depth doses at 0.5, 1, 5, 10, 15 and 20 cm water depths for 23 patient fields with head and neck cancers who were treated by a Cobalt unit. All 23 radiation fields were shielded by Cerrobend and lead blocks. (figure 1)

**Table 1:** Mean ±SD Percent Depth Doses from 0.5 to 20cm Water Depths, for 23 Radiation Fields Shielded by Lead and Cerrobend Blocks**

***Depth*** ** (*cm*) **	***PDD*** ** (*%*) ** ***Lead***	***PDD*** ** (*%*) ** ***Cerrobend***	***P,value***
0.5	100±0.00	100±0.00	-
1	98.97±.68	99.12±.64	0.45
5	80.92±2.48	80.67±2.39	0.72
10	58.08±2.89	58.4±3.17	0.72
15	41.52±2.88	41.5±3.36	0.98
20	29.55±2.58	29.44±3.02	0.8

Absolute dose means at 1.5- 3- 5, 7, 10, and 12cm water depths for 23 field sizes shielded by Cerrobend and lead blocks are shown in Table 2. Table 3 shows dose means at 0.5 and 1.5 cm solid water depths measured by TLD dosimeter for 4 head and neck field sizes shielded by Cerrobend and lead blocks.

**Table 2:** Mean ± SD of Absolute Doses at 1.5, 3, 5, 7, 10 and 12 cm Depths in Mini-Water Phantom for 23 Patient Field Sizes, SSD = 80 cm.

***Depth*** ** (*cm*) **	***Absolute Dose(cGy)*** ***Lead***	***Absolute Dose(cGy)*** ***Cerrobend***	***P,value***
1.5	113.98±23.58	114.30±23.29	0.96
3	105.42±20.66	105.73±20.98	0.96
5	93.33±18.5	93.42±16.92	0.98
7	81.35±14.4	81.85±14.23	0.91
10	66.64±10.84	67.17±10.73	0.86
12	58.02±10.13	58±9.61	0.99

**Table 3:** Mean ±SD of Doses at Surface, 0.5 and 1.5cm Solid Water Depths for 4 Patient Field Sizes Measured by TLD Dosimetery and Shielded by Lead and Cerrobend Blocks, SSD = 80 cm

***Depth*** ** (*cm*) **	***Absorbed dose*** ***Lead***	***Absorbed dose*** ***Cerrobend***	***P,value***	***Difference*** ** (*%*) **
Surface	111.96±7.69	109.13±6.31	0.26	2.53±1.63
0.5	115±7.07	114.08±6.26	0.16	2.05±0.86
1.5	108.91±6.67	107.76±6.88	0.63	1.09±.25

Mean Skin Sparing Effect (SSE) as the ratio of maximum depth dose to skin dose for 23 patient field sizes shielded by Cerrobend or lead blocks were 1.61±0.13 and 1.59±0.13, respectively.

SSE = D_max_ / D_skin_

## Discussion

The aim of this study was to compare the effects of lead and Cerrobend shielding blocks on the central axis dose of γ-beams of a Cobalt unit for different patient field sizes. 

In a report by Mohammadi et al, it was recommended that dose distribution in field sizes shielded by Cerrobend be assessed, regardless of Cerrobend advantages[10].

Iftekhari et al. reported that effects of field size, beam energy and shield size on the beam output had almost the same pattern for both lead and Cerrobend shielding blocks. Shield factors for determination of precise patient dose for all field sizes, beam energies and shield sizes were proposed[11].

Influence of shielding blocks on the output of a Cobalt unit and seven various accelerators were investigated by Dam et al. The loss in output due to shielding blocks was calculated, taking into account the loss in phantom scatter only. Reported experimental results showed that calculation algorithm is correct in most clinical conditions. However, for the blocks close to central beam axis, an overestimation of the output through algorithm was found[12]. When a block and tray are placed in an x-ray beam, the dose to a point in a phantom is changed by the following factors; (1) attenuation of photon and electron flow from the head of the accelerator by tray and block; (2) scatter decrease in phantom due to reduction of the volume of phantom receiving radiation and (3) generation of scatter off the tray and block. The third factor is generally ignored in dosimetry calculation. The scatter off a block could increase incident photon flow to 2%. The amount of this block scatter depends on the length of the inner edge of the block and block size which is irradiated. Total block–tray factor can be as much as 3% more than single-value tray factor which was measured for 10×10 cm field size[13]. Results of this study showed no significant difference between Cerrobend scattering and lead shielding blocks in different water phantom depths in clinical conditions. Cerrobend block shields are made divergent; therefore, they produce lower penumbra compared to standard lead blocks shielding. As a result, normal tissues around tumor received a lower dose. This property along with their easier use, saving time and accuracy in fabrication of the shield are advantages of using Cerrobend shield block instead of lead standard block. Furthermore, dosimetry parameters such as PDD and absolute doses at different depths under the field shielded by these two methods in clinical conditions exhibited no significant differences. These results are consistent with a report by Buenfil et al, in which “dose values measured by TLD for fields blocked by lead and Cerrobend blocks were statistically consistent”[14]. They showed a general tendency to lower values when lead blocks were used because lead blocks were 8.2 cm in thickness, but Cerrobend blocks were 7.6 cm in thickness and lead density was 1.2 times more than Cerrobend density; therefore, for a given Cerrobend thickness, a more radiation transmission would be expected[14]. In this study, Cerrobend thickness was almost similar to that of lead block, i.e. 4.6 and 5 cm, respectively (suitable for a Cobalt unit).

## Conclusion

Based on the results of this study and comparisons made between present study and some previous studies, the use of Cerrobend shields apart from advantages such as saving time, reproducibility, decrease in penumbra size, and precision, accuracy in shield fabrication dose not exhibit any significant differences in isocenter doses compared to block shields; therefore, it is recommended that Cerrobend shields be used in radiotherapy centers. 
